# Rooting mediates the effect of stress by acculturation on the psychological well-being of immigrants living in Chile

**DOI:** 10.1371/journal.pone.0219485

**Published:** 2019-08-13

**Authors:** Alfonso Urzúa, José Leiva-Gutiérrez, Alejandra Caqueo-Urízar, Pablo Vera-Villarroel

**Affiliations:** 1 School of Psychology, Universidad Católica del Norte, Antofagasta, Chile; 2 Department of Psychology & Philosophy, Universidad de Tarapacá, Arica, Chile; 3 Universidad de Santiago de Chile, Santiago, Chile; Middlesex University, UNITED KINGDOM

## Abstract

Migration is a social phenomenon that has an impact both on the lives of the people who migrate, and on the societies who receive them; with psychological well-being being one of the most affected variables. The objective of this research is to analyze the possible mediating role of rooting in the host location on the negative effect that acculturation stress has on the level of well-being. Data for this study were collected using 699 Colombian and Peruvian immigrants who have been permanently residing in Chile for more than six months. Participants were assessed by using Riff’s Psychological Well-being Scale, rooting of Torrente et al., and Ruiz et al. scales of stress. The results demonstrated the mediating role of settling down within the host country in relation to stress and psychological well-being, except for the sub-dimension of autonomy. It is concluded that the need for rooting in the host country is a protective factor against the negative effects of stress on perceived well-being.

## Introduction

Psychological well-being is an eudiamonic indicator of well-being linked to aspects of positive functioning, such as purposeful engagement in life, realization of personal talents and capacities, and enlightened self-knowledge. [1, 2, 3], or to a state of equilibrium and balance that can be affected by life events or challenges of life itself [4]. Although measures of well-being tend to be stable over time [5, 6], they can be affected by intense transitory circumstances or by alterations in the context a person’s daily life. One of these events is that of immigration.

Immigration can be understood as the movement of people from one country to another in order to improve personal, social, or material conditions [7]. This phenomenon is present in many countries of the world, and Chile is no exception. In 2014, the immigrant population in Chile represented 2.3% of the total population [8], increasing to 4.35% in 2017 [9].

This movement has different consequences for immigrants who, faced with a different culture, must undergo a series of internal transformations to adapt [10]. However, there are situations in which the changes demanded by the host culture are superior to the capacities of the individuals who must face them, giving rise to what is known as stress by acculturation [11].

Even though immigration does not necessarily constitute a stressor, the conditions in which it occurs can trigger acculturation stress. When this occurs, it can have negative consequences on the mental health and well-being of the immigrant population [10, 12, 13]. Considering the importance of well-being in people’s lives, it is particularly relevant to investigate not only the elements that could affect well-being, but also the extent to which other variables could, both negatively and positively, explain it [14].

One element that emerges from this scenario, and that could have a positive effect on well-being, the feeling of being a member of the host country. This constitutes a rooting, defined as the process through which a particular relationship with a region is established, creating ties that maintain the link with the locality for different reasons [15]. Other authors add the feelings of attachment or detachment that humans experience and express in relation to a specific locality [16] to constitute a sense of belonging.

In simple terms, it would be equivalent to the expression "To throw roots" -from Spanish *echar raices*-. Thus, when applying this definition to the context of immigration, it would refer to the link formed with the location of reception; thereby constituting an important element in enhancing psychological well-being [17], becoming an important consideration for determining physical and psychological well-being [18].

This process of developing a rooting to the host country may be influenced by the context of the country itself. Thus, if there is stress due to acculturation, that is, if the process of adapting to the host culture exceeds the person’s abilities to acculturate, it will be difficult to feel part of the host culture. For example, feeling discriminated by the host culture negatively affects both mental health and well-being, thus making it difficult to feel part of the culture that is discriminating against it [14,19]. For this reason, it can be hypothesized that the immigrant experiences the negative effects of stress due to acculturation when settling-in to the host country.

Thus far, it has been possible to mention the effect that two variables have on well-being. Firstly, the stress of acculturation as an element that would diminish well-being, and, at the same time, the positive effect of feeling part of the host country, which could assume a mediating role in the relationship between stress due to acculturation and psychological well-being. Considering the, this research aims to evaluate the mediating role of settling down within the host country in the relationship between acculturation stress and the psychological well-being of Latin American immigrants living in Chile. It is expected that not only is there a direct relationship between stress and well-being, but that in turn, this relationship is effectively mediated by being rooted in the host country.

## Method

### Participants

In this study, data were collected from a total of 699 Peruvian and Colombian immigrants, both men and women, over 18 years of age, and who have been residing in Chile for longer than six months, regardless of their legal status in the country. Immigrants from three cities, who were contacted by combined snowball and intentional sampling, participated. Fifteen questionnaires were not completely answered, so they were eliminated from the analyzes, leaving, therefore, the final sample constituted by 684 participants Of the participants, 48.2% (330) were men and the remaining 51.8% (354) were women, with ages between 18 and 71 years, with an average age of 33.19 (SD = 9.54). Colombian immigrants accounted for 53.1% (363) and 46.9% were Peruvian (321). Regarding their legal status, 434 (63.5%) reported having permanent residency, 220 (32.2%) did not have permanent residency, and 29 (4.2%) did not require it since they were nationalized.

### Instruments

#### Ryff’s Psychological Well-being Scale (1989)

A self-report questionnaire adapted by Van Dierendonck, [20] and translated into Spanish by Díaz, et al. [21] allowed for the evaluation of the different domains of psychological well-being, using a Likert type scale, ranging from 1 (*totally disagree*) to 6 (*totally agree*). It is composed of 29 items, which were subdivided into six subscales: self-acceptance (feeling good about oneself, considering one’s limitations), positive relationships (stable and trustworthy friendship relationships on which to count on), autonomy (ability to sustain one’s own individuality in different social contexts), domain of environment (ability to choose or create favorable environments for oneself), purpose in life (possession of clear goals and ability to define goals and vital goals), and personal growth (capacity to generate conditions that allow for the development of potentialities). To interpret, obtaining higher scores on the subscales indicates greater subjective well-being.

The reliability indexes of the instrument have been shown to be apt, both in the original version proposed by Van Dierendonck [20] and in the other studies carried out in Chile [22–24]. Similarly, this instrument has been used on several occasions in Chile, in different populations including immigrants, with adequate psychometric properties [14, 25, 26].

**Fig 1:**
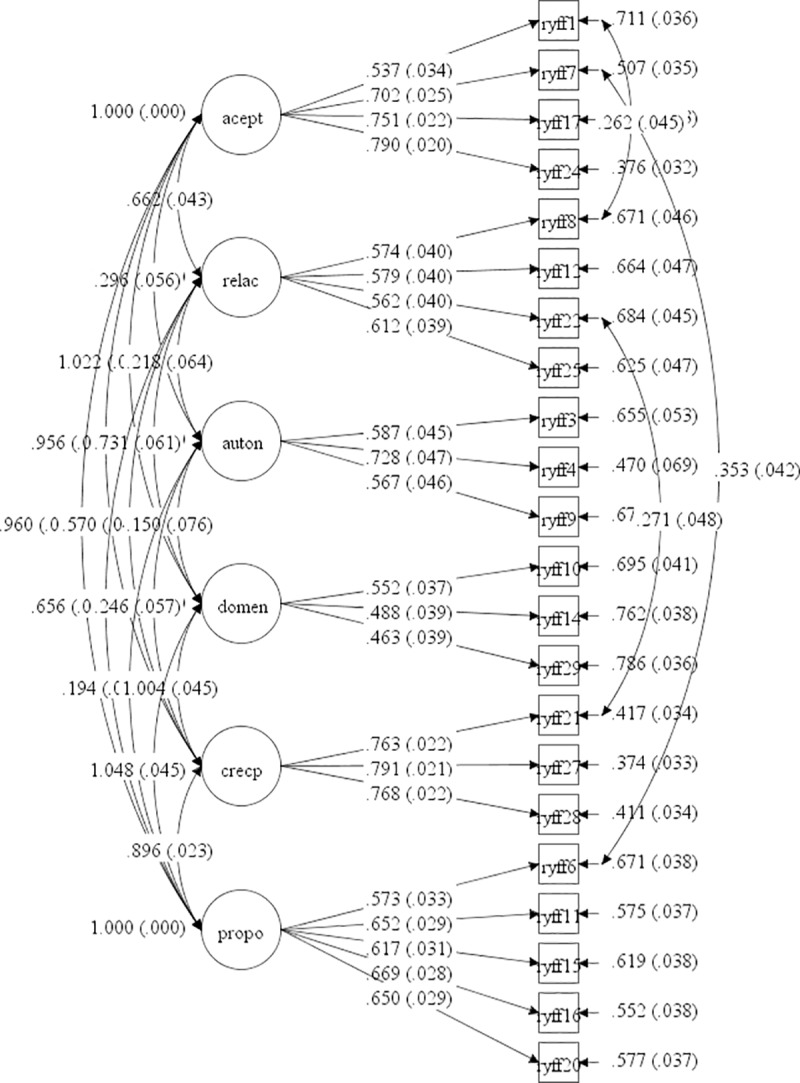
Measurement model for psychological well-being scale. Confirmatory factor analysis results using SEM are provided.

An analysis of the measurement model of the dimensions that make up the scale was made. The items that had very low saturations and high errors were eliminated. In the final model, a CFI = .926 and an RMSEA = .057 I.C.90% [.051 - .063] were obtained (Fig 1).

#### Stress by acculturation

The scale proposed by Ruiz, Torrente, Rodríguez, and Ramirez [27] was used. It is a scale of 24 items with a Likert format, ranging from 0 (*I have not had this problem*) to 5 (*it has greatly affected me*). This scale considers six dimensions of stress: perceived discrimination, differences with the out-groups, problems of legal status, problems in social relations with other immigrants, and distance from the home and broken families. In turn, for reasons of parsimony, we estimated a single indicator of stress by acculturation, which is used in this study.

This scale obtained adequate validity and reliability indexes, both in its validation study [27] and in studies conducted in Chile using this instrument [10, 13, 28].

In order to analyze the measurement model, an exploratory factor analysis was carried out with a random subsample of 200 subjects, using the Maximum Likelihood extraction method with a Direct Oblimin rotation.

In the first factorial solution, 3 dimensions of the originally proposed 6 were obtained. Thus, the family rupture domain was not satisfied, since the items 13 and 22 that compose it did not saturate in any domain. Item 18 also had no saturations greater than .30.

A second exploratory analysis was carried out without considering these reagents, where factor 1 grouped items of the dimension Discrimination and perceived rejection (“*some Chileans give me to understand that this is not my country*”). Factor 2 grouped the dimensions of Problems of citizenship and legality (“*They have abused me at work for being an immigrant*”). Factor 3 grouped the items of the Distance dimension with the origin (“*feel away from my family*”) plus an item of Differences with outgroup (item 14). In factor 4, the items of Problems of social relations with other immigrants (“there are compatriots who take advantage of me”) were saturated in addition to an item of the Differences with outgroup dimension (item 8). Finally, factor 5 grouped items originally from the Differences with outgroup dimension (items 20 and 23), a Distance item with the origin (item 4) and three items of Discrimination and perceived rejection (items 24, 9 and 21). Given the scattered theoretical content of the questions that saturated in factor 5, it is decided to eliminate this dimension.

**Fig 2:**
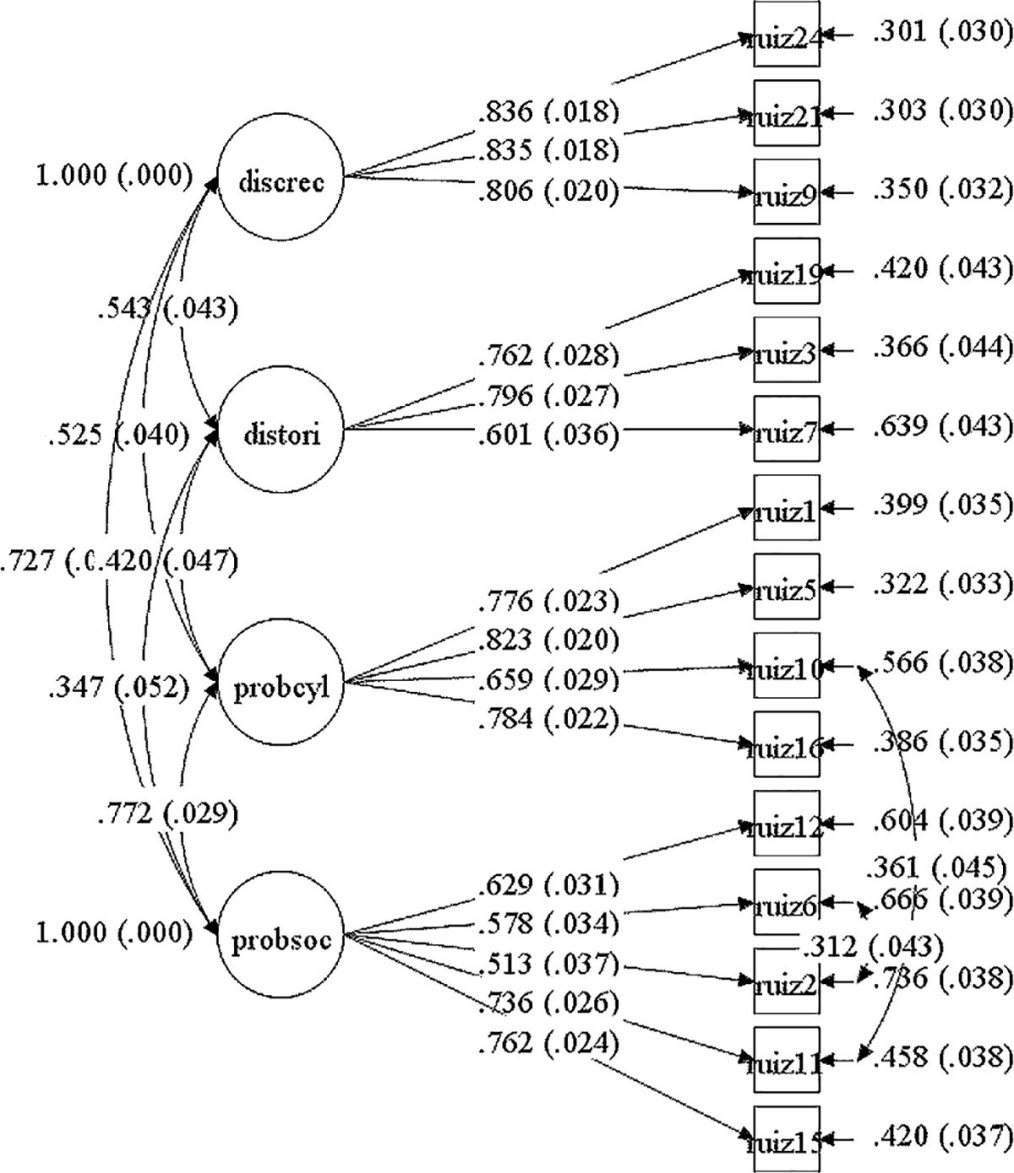
Factorial diagram for acculturation stress scale. Confirmatory factor analysis results using SEM are provided.

Subsequently, a confirmatory factorial analysis was carried out considering the four factors mentioned (CFI = .933, RMSEA = .076 IC90% [.067 - .085]) and one considering these same factors plus a second order factor (CFI). = .916, RMSEA = .084 IC90% [.076 - .092]). Finally, the global adjustments of both models were compared, concluding that the model with a second order factor has a significantly lower adjustment (Table 1), thus using the 4-factor model for the following analyzes (Fig 2).

**Table 1:** Comparison of nested models for acculturation stress scale. Chi squared difference was used to compare both 4 factors and 4 factors and a second order factor models. Inferential statistics are provided.

Model	Chi squared	GL	p	Dif. Chi squared	Dif DF	p
4 factors	386.183	84	.00	–	–	–
4 factors +1 second order factor	324.139	82	.00	62.044	2	.00

#### Rooting within the host country

The instrument was created by Torrente, Ruiz-Hernandez, Ramirez, and Rodríguez [29]. It is composed of 16 questions with a Likert format, ranging from 1 (*nothing at all*) to 5 (*much*). It considers three dimensions that make up a unique indicator of settling down: cultural roots (“feels Chile's social customs as its own”), ecological roots (“feels the city in which he lives as his own”), and social roots (“he feels linked to his co-workers”). In the creation of this scale, it obtained adequate reliability and validity indexes [29].

**Fig 3:**
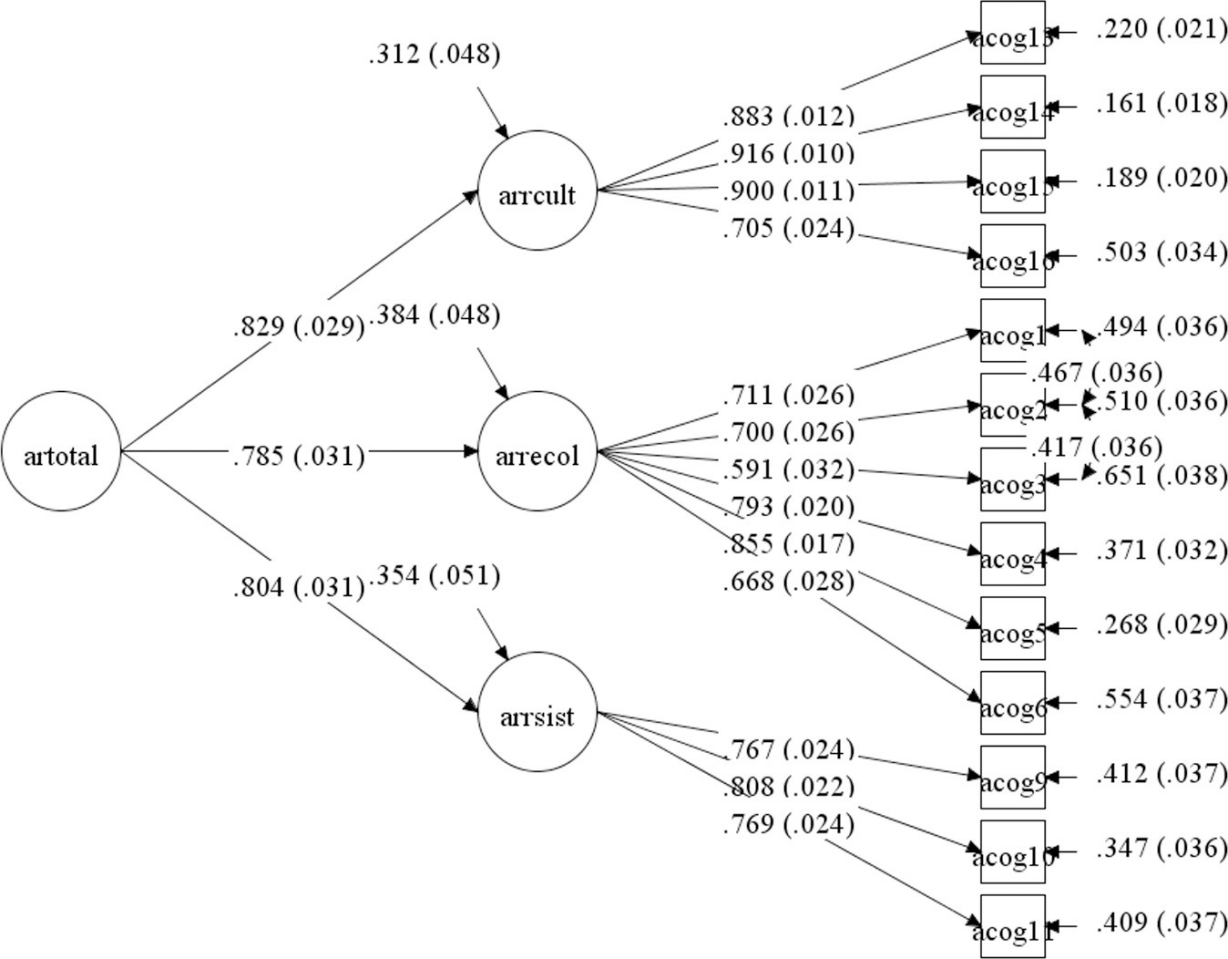
Measurement model for rooting scale with host country. Confirmatory factor analysis results using SEM are provided.

A confirmatory factorial analysis was carried out based on the three theoretical factors, comparing goodness of fitness indicators against a model of a second-order factor that showed the rooting with the host country in a global manner. For the three-dimensional model we obtained a CFI = .977 and an RMSEA = .056 IC90% [.046 - .067], which are in appropriate ranges, values ​​similar to that found in a second order factor model, using this last model in the subsequent analyzes by parsimony effect (Fig 3).

### Procedures

The data collection began after obtaining the approval from the Ethics Committees of the Universidad Católica del Norte and the National Commission of Science and Technology. Each participant was informed about the objectives of the project, for later, if they agree to participate, sign a written consent, leaving a copy in their possession. Given that the size of the population universe was unknown, a sampling technique was used that combined the snowball technique with intentional sampling in order to comply with the quotas of immigrants from different countries and cities of residence [30]. In order to ensure the representativeness and guarantee the diversity of the participants, five different initial samplings were made of the data in different cities of Chile (Arica, Antofagasta, and Santiago).

The data was tabulated in a database using the IBM SPSS statistical software. v.21. Descriptive statistics analysis of the dimensions of interest was carried out. Subsequently, the Mplus v.7 program was used in order to perform the mediation analysis required to evaluate the proposed model.

## Results

### The effect of mediation

The effect of the mediating variable on the indicators of well-being was studied in order to later study the total, direct, and indirect effect of stress on the same indicators.

Below, the direct, indirect and total effects are detailed. First, these are shown in a summary table (Table 2) and then the respective diagrams. For parsimony, the diagrams do not show the saturations of the items.

**Table 2:** Direct, indirect and total effects. Indirect (IE), direct (DE) and total efects (TE) of acculturation stress on psychological wellbeing are provided.

	Discrimination and rejection perceived			Distance to the origin			Problems of citizenship and legality			Problems of social relations with other migrants		
	DE	IE	TE	DE	IE	TE	DE	IE	TE	DE	IE	TE
Self-acceptance	.108	-.015	.042	.244*	-.006	.126*	-.066	-.023	-.058	-.494*	.045	-.364*
Positive relationships	.079	-.050	.000	.051	-.020	.013	.032	-.080*	-.060	-.400*	.156*	-.243
Autonomy	-.027	.015	-.004	-.020	.006	-.009	.143	.023	.121	-.419*	-.046	-.506*
Domain of the environment	.138	-.023	.058	.291*	-.009	.166*	-.179	-.036	-.140	-.460*	.071	-.354*
Personal growth	.081	-.013	.034	.281*	-.005	.163*	-.143	-.021	-.104	-.344*	.042	-.275*
Purpose in life	.075	-.014	.025	.293*	-.006	.152*	-.188	-.023	-.120*	-.358*	.044	-.252*

*p < .05, DE = Direct Effectt; IE = Indirect Effect; TE = Total Effect

**Fig 4:**
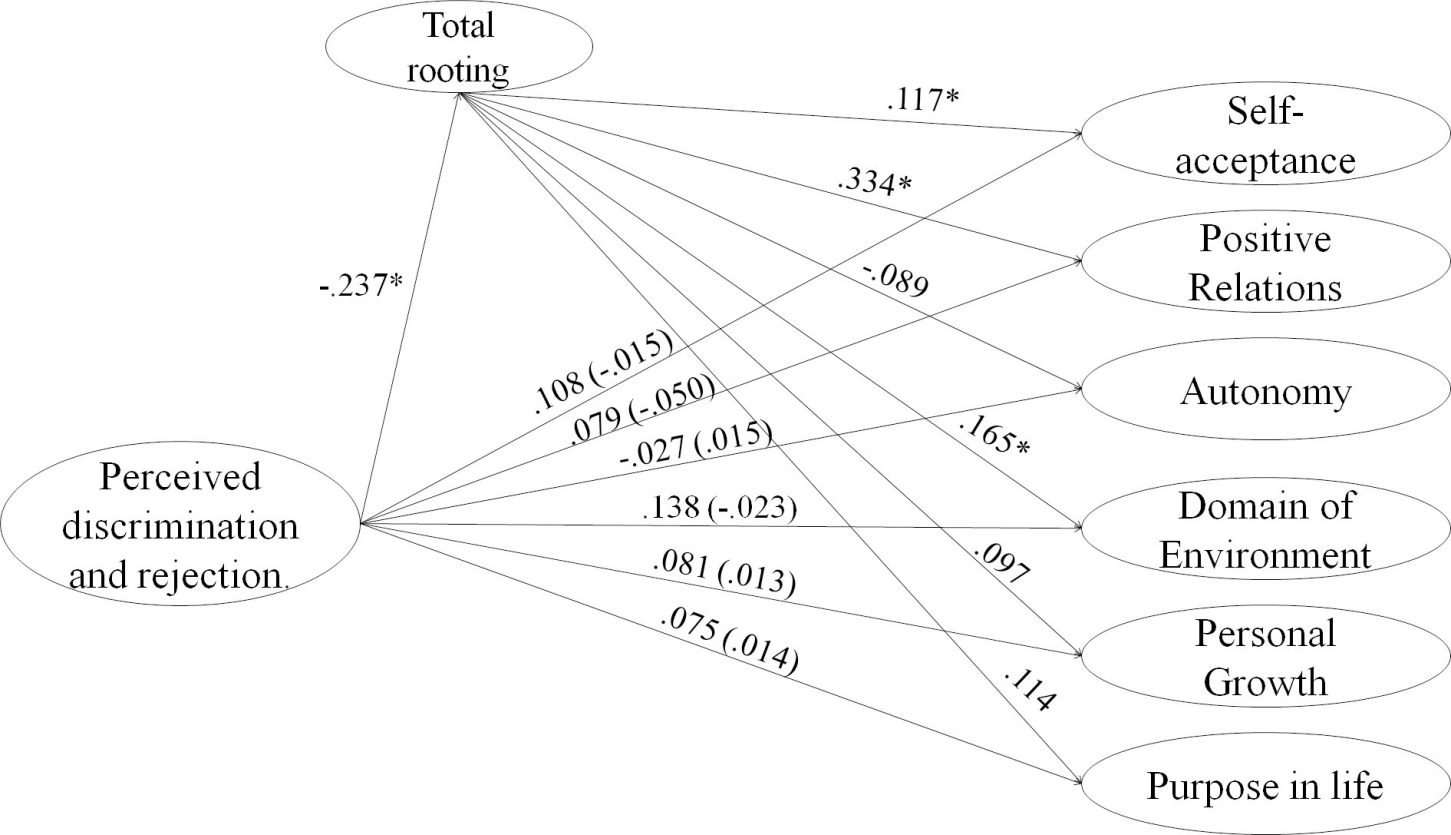
Structural model for the mediating effect of rooting with the host country in the relationship between perceived discrimination and psychological well-being. Indirect, and direct effects are shown on this figure for perceived discrimination as independent variable.

**Fig 5:**
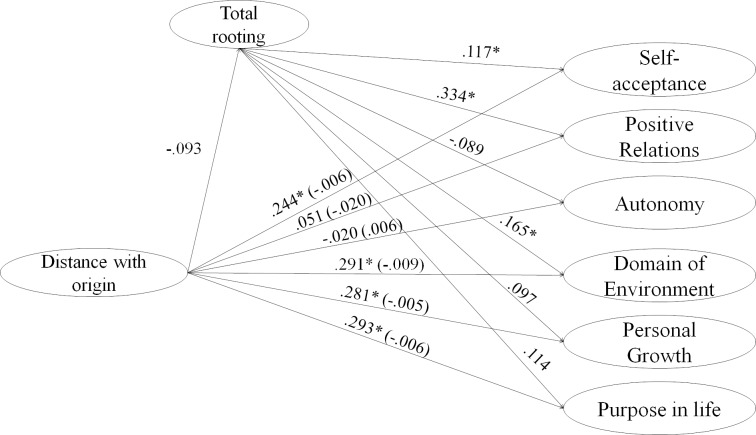
Structural model for the mediating effect of rooting with host country in the relationship between distance difficulties with origin and psychological well-being. Indirect, and direct effects are shown on this figure for distance difficulties with origin as independent variable.

**Fig 6:**
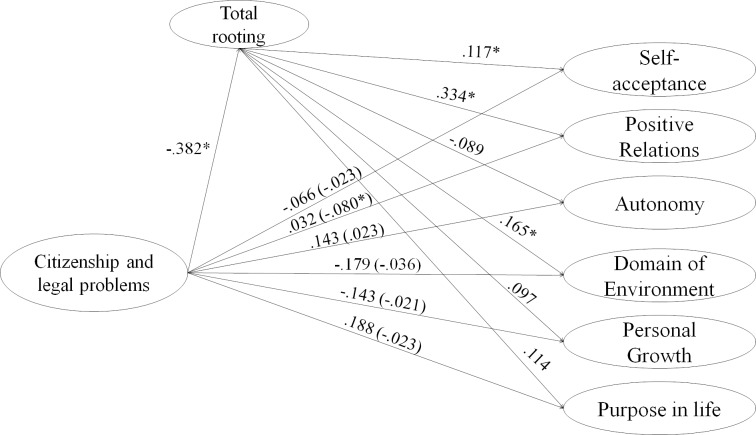
Structural model for the mediating effect of rooting with the host country in the relationship between legal problems and citizenship and psychological well-being. Indirect, and direct effects are shown on this figure for legal problems and citizenship as independent variable.

**Fig 7:**
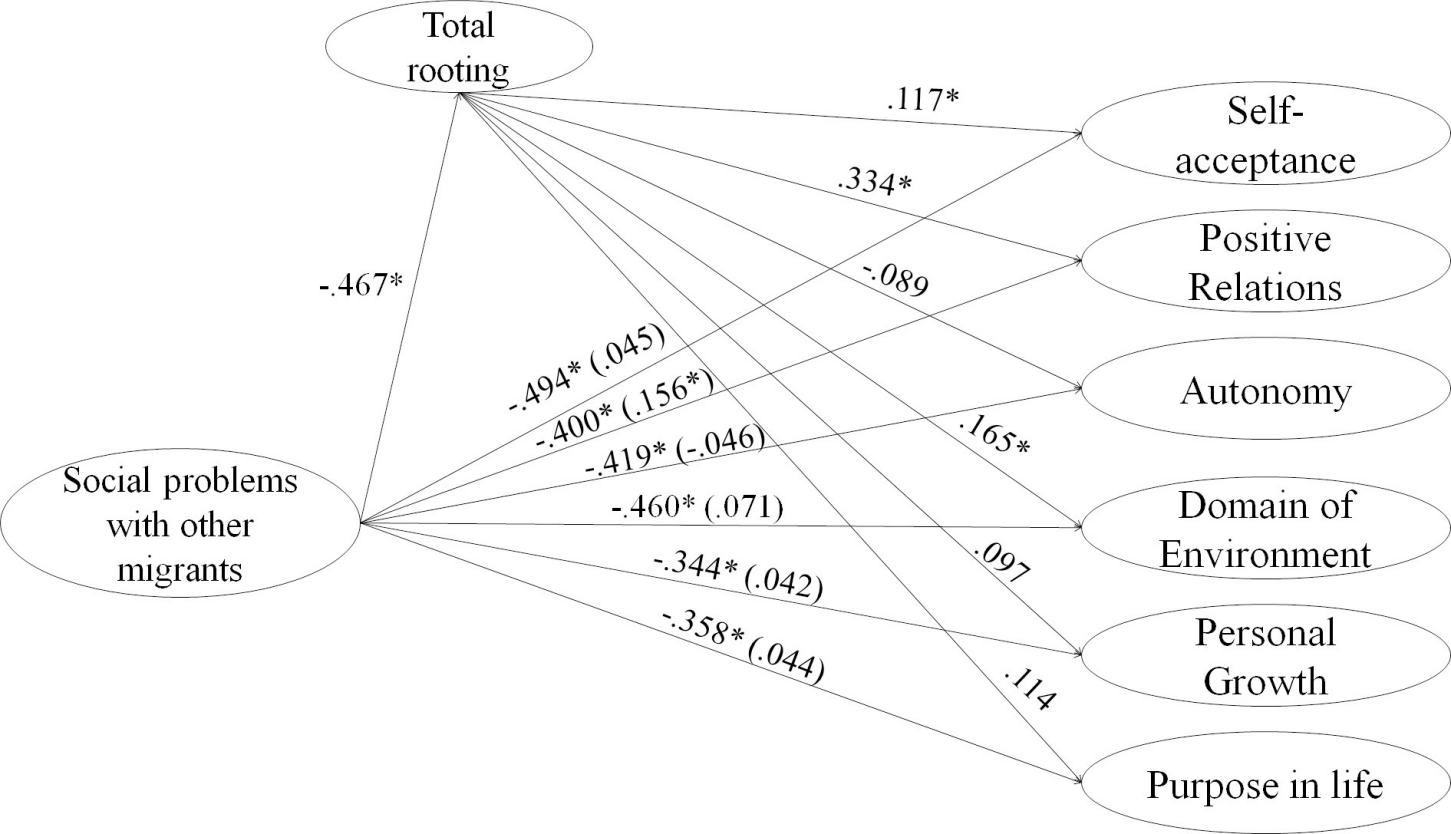
Structural model for mediating effect of rooting with the host country in the relationship between social problems in the relationship with other migrants and psychological well-being. Indirect, and direct effects are shown on this figure for problems with other migrants as independent variable.

The structural model obtained a CFI = .922 and an RMSEA = .042 I.C 90% [. 039 - .044]. Figs 4–7 show the simple and indirect effects between the independent, dependent and mediating variables. To simplify the appreciation of the model, four figures will be presented, one for each of the dimensions of the independent variable. However, it is important to note that it is a single model.

Regarding the direct effects of the independent variable, it is found that the variables Discrimination and Rejection perceived, and problems of citizenship and legality had no relationship with the dimensions of well-being as can be seen on Figs 4 and 6. On the other hand, Distance with the origin was negatively related to the domain of the environment (Fig 5), Personal Growth and Purpose in life, while the effect was positive on Self-acceptance. Finally, social problems with other migrants were negatively related to all dimensions of psychological well-being (Fig 7).

Second, all dimensions of Stress by acculturation had a significant and negative effect on the proposed mediator except Distance to the country of origin (Figs 4–7). On the other hand, the rooting with the host country had an effect only on the Self-acceptance, the positive Relationships and the Domain of the environment.

Finally, regarding the indirect effects, rooting had a mediating effect only on the effects of Problems of citizenship and legality and Problems with other migrants on the positive relations of well-being dimension.

In this way, rooting partially mediated the effects of stress due to acculturation and psychological well-being.

## Discussion

The objective of this research was to identify the mediating effect of rooting with the host country on the relationship between stress for acculturation and well-being.

The results show the mediation of the rooting in the effect of two of the dimensions of stress by acculturation on positive relationships.

The fact that rooting with the host country, that is, feeling part of that country, mediated the effect of stress on the social component, suggests that the relational aspect is especially relevant in feeling part of the country. This can also be sustained given that the greatest effect was precisely on this dimension.

For the same reason, it is suggested to investigate other intrapsychic variables that allow to better explain the effect that stress generates on well-being. Although the effect of stress was not fully mediated by rooting, rooting had indeed a direct effect on three of the six dimensions of psychological well-being. This highlights the role of this variable as an important factor to consider when working with migrant populations and their acculturation´s stress.

An interesting element is the relationship between stress by acculturation and rooting with the host country. As hypothesized in the introduction, the fact that stress by acculturation is related to the environment demanding a level of high adaptation to what the person is capable of [10], may imply that the person Do not feel part of the place because you cannot meet these requirements. In fact, this relationship with the host country could tend to be negative, with negative feelings towards it, according to the definitions explored [16]. For this reason, it was expected that the effect of stress on the rooting was negative, which was effectively fulfilled in all dimensions except for the difficulties caused by distance. That is, the distance did not affect the feeling of belonging to the host country, but the rest of the dimensions, which implies considering as a relevant antecedent in the fact of feeling part of the country, the different legal or citizenship difficulties, discrimination and conflicts with other migrants.

Considering the above, the work with migrant people becomes relevant when there are the aforementioned difficulties to adapt to the host country or when the demands of adaptation surpass individuals, especially when they are problems with other migrants and product of distance (which are the dimensions that had an effect on welfare). With this research it was possible to provide a background that would serve to guide this work considering the dimensions of well-being that are affected by stress.

On the other hand, it was possible to identify the role played by feeling part of the country of origin, placing it as an important element to consider in working with migrants. When intervening the stress by acculturation, or directly the psychological well-being of the subjects, one should have as a relevant element to consider that they feel part of the place that welcomes them, so that this allows to lessen the effect that the stress will have on the health mental. Although he only mediated in part the effect of stress on positive relationships, it did have an effect on three dimensions of well-being. Therefore, it cannot be left aside when intervening in groups of foreigners residing in the country.

Finally, it is important to mention that the level of roots in both Peruvian and Colombian population was not significantly different, but some indicators of stress due to acculturation. Given that the Colombian participants had higher averages, it is also that this group should be considered to intervene. One of these dimensions that resulted with a higher average was the perceived discrimination, which to be changed requires not only an intervention with the migrant group but with the host population.

This study is a first approach and its limitation is that it was not possible to carry out a longitudinal follow-up of the participants. However, since it was possible to access a large number of people, it was possible to perform analyzes that allowed explaining most dimensions of well-being.

In the future it is proposed to investigate other variables that explain well-being in order to have a broader view of those factors that affect this important topic of people's lives. Likewise, it is proposed to study in more detail the variables that explain the autonomy of people, since although it was found to be related to stress, it is not explained by the proposed mediator (unlike the other dimensions of well-being).

## Supporting information

S1 Data S1_data.DAT. Data used for the analysis with software Mplus.
(DAT)
