# Correction: Rooting mediates the effect of stress by acculturation on the psychological well-being of immigrants living in Chile

**DOI:** 10.1371/journal.pone.0241873

**Published:** 2020-10-29

**Authors:** Alfonso Urzúa, José Leiva-Gutiérrez, Alejandra Caqueo-Urízar, Pablo Vera-Villarroel

This article [1] did not list the correct and current affiliation information for the fourth author. Pablo Vera-Villarroel was affiliated with Universidad de Santiago de Chile when this study's data were collected and when the manuscript was written, but he was no longer affiliated with this university at the time of the article's publication. Pablo Vera-Villarroel is currently affiliated with Centro Análysis I+D, Santiago, Chile.
